# The Impact of COVID-19 Lockdown Measures and COVID-19 Infection on Cognitive Functions: A Review in Healthy and Neurological Populations

**DOI:** 10.3390/ijerph20064889

**Published:** 2023-03-10

**Authors:** Alessio Manfredini, Francesca Pisano, Chiara Incoccia, Paola Marangolo

**Affiliations:** 1 Department of Humanities Studies, University Federico II, 80133 Naples, Italy; 2 IRCCS Fondazione Santa Lucia, 00179 Rome, Italy

**Keywords:** COVID-19 infection, confinement measures, lockdown, standardized cognitive tests, cognitive deficits

## Abstract

The COVID-19 pandemic severely affected people’s mental health all over the world. This review aims to present a comprehensive overview of the literature related to the effects of COVID-19 lockdown measures and COVID-19 infection on cognitive functioning in both healthy people and people with neurological conditions by considering only standardized tests. We performed a narrative review of the literature via two databases, PUBMED and SCOPUS, from December 2019 to December 2022. In total, 62 out of 1356 articles were selected and organized into three time periods: short-term (1–4 months), medium-term (5–8 months), and long-term (9–12 months), according to the time in which the tests were performed. Regardless of the time period, most studies showed a general worsening in cognitive performance in people with neurological conditions due to COVID-19 lockdown measures and in healthy individuals recovered from COVID-19 infection. Our review is the first to highlight the importance of considering standardized tests as reliable measures to quantify the presence of cognitive deficits due to COVID-19. Indeed, we believe that they provide an objective measure of the cognitive difficulties encountered in the different populations, while allowing clinicians to plan rehabilitation treatments that can be of great help to many patients who still, nowadays, experience post-COVID-19 symptoms.

## 1. Introduction

The coronavirus disease 2019 (COVID-19), caused by the severe acute respiratory syndrome coronavirus 2 (SARS-CoV-2), is a global epidemic that is still circulating across countries, leading to public health crises throughout the world [1,2,3]. To contain the speed of viral transmission, many national governments enacted different restrictive measures, such as social distancing, face coverings, avoidance of crowded places, testing, and tracing [4,5,6]. These measures were first limited to the most affected areas, but were rapidly extended to entire countries worldwide [7,8,9]. Regulations also consisted of lockdown measures aimed at further reducing exposure to contagion, which were implemented by the central and local authorities in different ways in China, European nations (such as Italy and Spain), and in the United States [10,11]. However, despite the active vaccination campaigns still in progress worldwide, it is difficult to achieve global control of the pandemic [6].

As it has been now well-documented, lockdown measures and infection due to COVID-19 have greatly affected people’s mental health resulting in severe psychological and cognitive consequences [12,13,14,15]. Indeed, higher levels of anxiety, depression, and stress have been recorded during the confinement period compared to the pre-COVID-19 emergency, disrupting the balance of daily activities and the perception of well-being in both healthy people [16,17,18,19,20] and people with neurological conditions [21,22,23,24]. Lockdown measures imposed during the COVID-19 pandemic also caused cognitive changes in different populations [20,25,26,27,28]. For instance, in Nogueira et al.’s study [28], a deterioration of cognitive flexibility and processing speed compared to pre-COVID-19 confinement was detected in a group of healthy subjects. Additionally, subjective cognitive decline complaints also significantly increased during the pandemic [28]. During COVID-19 lockdown, Pisano et al. [20] reported a decline in working and prospective memory assessed on standardized cognitive tests in a sample of young university students. At the same time, Baschi et al. [25] described a worsening of cognitive, behavioral, and motor symptoms in Parkinson’s (PD) and Mild Cognitive Impairment (MCI) patients. The negative impact of COVID-19 isolation on cognitive functioning was also reported by Chen et al.’s study [27]. In their study, Alzheimer’s (AD) and dementia with Lewy bodies (DLB) patients exhibited an accelerated cognitive decline and neuropsychiatric symptoms over a one-year follow-up period [27].

It is worth noting that 43% of individuals affected by COVID-19 infection, including asymptomatic cases, and approximately 80% of patients hospitalized due to COVID-19 may experience post-COVID-19 sequelae [29,30]. Fatigue and cognitive impairment, along with other enduring neuropsychiatric (e.g., depression) [31] and physical (e.g., dyspnea) manifestations, have been described as part of the ‘post-acute sequelae of SARS-CoV-2′ (i.e., symptoms persisting for at least four weeks following infection) [32], colloquially, also referred to as “long COVID” or “post-COVID” [33,34].

As for studies on COVID-19 lockdown, several studies have investigated the effects induced by COVID-19 infection on cognitive functioning in healthy and neurological populations [35,36,37,38] using either self-reported questionnaires or standardized tests.

In a New York cohort of 740 COVID-19 patients (50% managed in a community setting), Becker et al. [35] reported a deterioration in memory encoding (24% affected), category fluency (20%), processing speed (18%), and executive functions (16%) [35]. A prospective study by Frontera et al. [37] showed that patients with neurological complications during index hospitalization had significantly worse six-months functional and cognitive outcomes than those without. Importantly, the authors found that approximately 50% of COVID-19 patients reported cognitive deficits and 47% was unable to return to work after six months. In line with this evidence, Boesl et al. [36] administered a screening test and self-questionnaires to a sample of 100 patients who presented with persisting neurological symptoms 12 weeks after the acute infection with SARS-CoV-2. The residual neurological symptoms indicated the persistence of fatigue, headache, and pathological scores on the Montreal Cognitive Assessment Scale, a test used by healthcare providers to evaluate the presence of cognitive decline [39].

Given the above reported results, the scope of this review is to present a comprehensive overview of the literature related to the effects of COVID-19 lockdown measures and COVID-19 infection on cognitive functioning in healthy people and people with neurological conditions. To this end, we decided to investigate only studies which used standardized tests to assess cognitive decline. Indeed, since self-reported questionnaires are more susceptible to social desirability and self-reported bias, they might lead to inaccurate self-reports and erroneous study conclusions.

## 2. Materials and Methods

### 2.1. Search Strategy and Selection Criteria

We conducted this study using the scope reviews methodological framework. We searched for articles on cognitive effects of COVID-19 lockdown measures and COVID-19 infection among healthy people and people with neurological conditions on two databases: PubMed and Scopus. Four different searches were conducted using different keywords combined with the Boolean operator “AND” and “OR”. The search period was set from December 2019 to December 2022. Keywords included: (COVID-19 lockdown or confinement measures) AND (Cognitive deficits OR Memory deficits OR Language deficits OR Attention Deficits); (Long COVID-19 OR Post COVID-19 OR Cognitive Sequelae of COVID-19) AND (Cognitive deficits OR Memory deficits OR Language deficits OR Attention Deficits); (Long COVID-19 OR Post COVID-19 OR Cognitive Sequelae of COVID) AND (Parkinson OR Dementia OR Alzheimer OR Stroke); (COVID-19 lockdown OR confinement measures) AND (Parkinson OR Dementia OR Alzheimer OR Stroke).

Included articles met the following criteria: (i) only studies using standardized cognitive tests on the effects of the COVID-19 lockdown/confinement measures and on the effects of COVID-19 infection among healthy people and people with neurological conditions; (ii) only studies conducted with participants over 18 years of age; and (iii) only studies with samples larger than 20 participants (N = >20); (iv) only studies conducted between December 2019 and December 2022. We excluded non-COVID-19 articles and COVID-19 articles not related to the study. Articles were also excluded if they were reviews, single case studies or case series. After eliminating duplicates, all potentially relevant full texts were screened by the authors (AM, FP) independently of one another to exclude non-eligible items.

### 2.2. Data Extraction and Analysis

**Figure 1:**
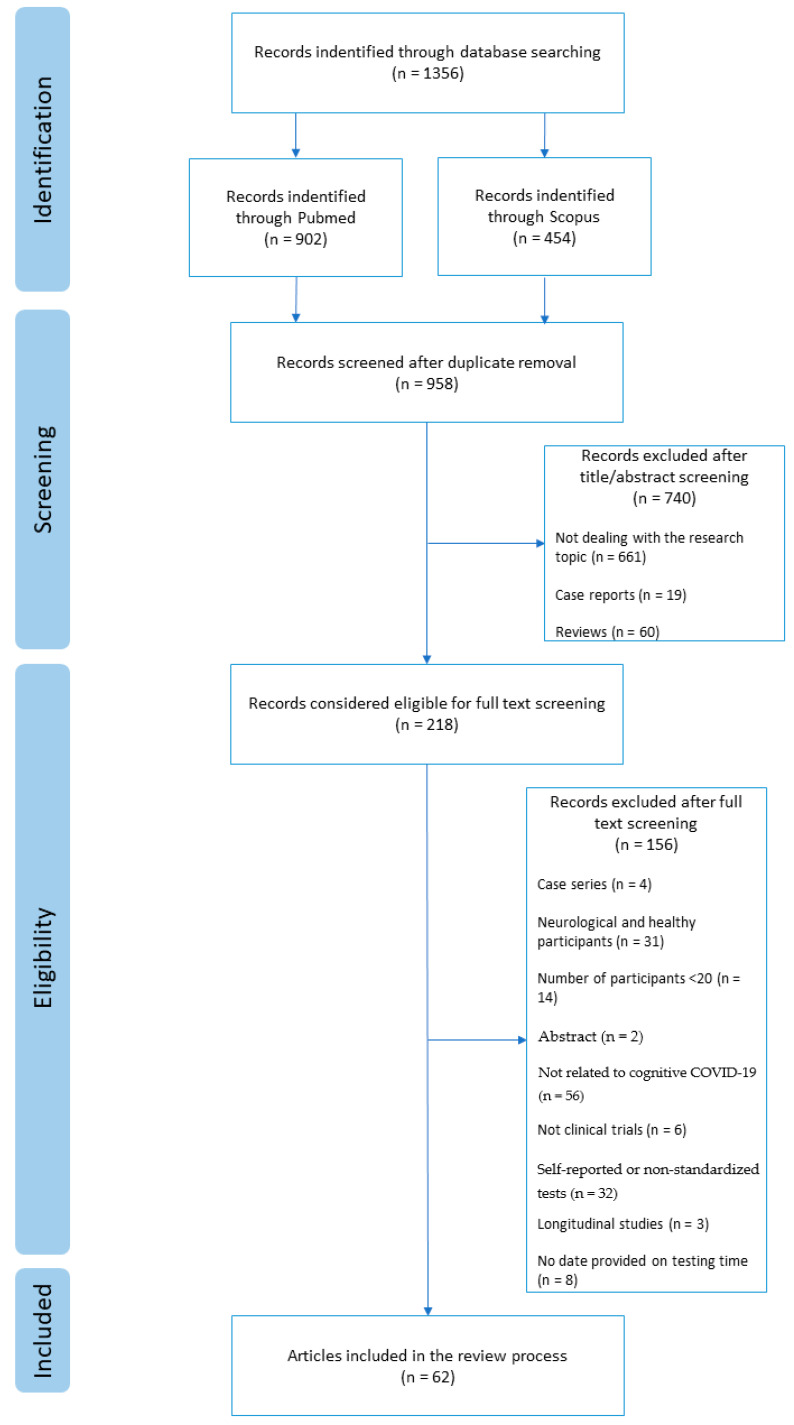
Flow diagram of review process.

A total of 1356 articles were retrieved through database searching. After the removal of 398 duplicates, a total of 958 articles remained, out of which 661 articles were excluded by title or abstract for not dealing with our research topic, 19 were removed as reporting case series, and 60 were excluded as referring to reviews. A total of 218 articles were considered eligible for the study. After full text screening, another 156 articles were removed since four were single cases, 31 mixed neurological with healthy participants, 14 included less than 20 participants, two had only the abstract available, 56 were not related to cognitive sequelae of COVID-19, six were not clinical trials, 32 did not include standardized tests, three were longitudinal studies, thus, it was not possible to individuate a precise period of testing time, and eight did not report the time of testing (see Figure 1).

**Figure 2:**
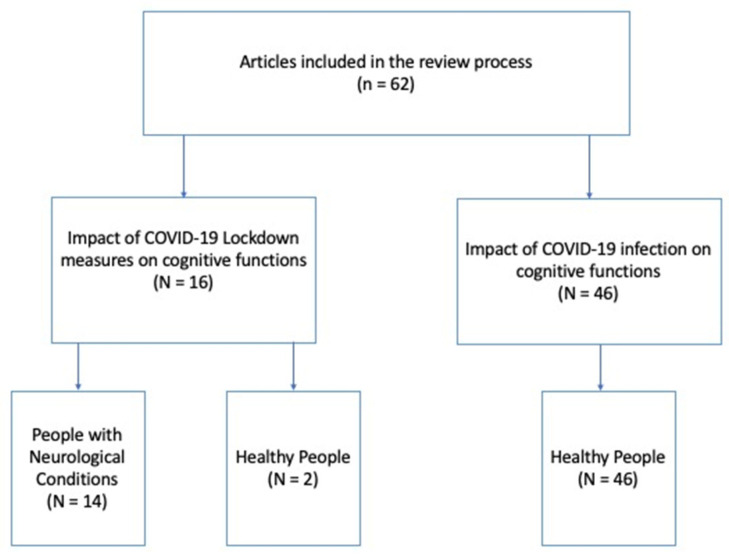
Articles included in the review process.

The selected 62 articles were rearranged according to the two principal aims of the review: (1) studies on the impact of COVID-19 lockdown measures on the cognitive functions (N = 16) of, respectively, (1a) people with neurological conditions (N = 14) and (1b) healthy people (N = 2); and (2) studies on the impact of COVID-19 infection on cognitive functions (N = 46). No studies on people with neurological conditions met our inclusion criteria in this category; thus, all studies in this category referred to healthy people (N = 46; see Figure 2). Finally, for each category, studies were organized into three further subgroups according to the time elapsed between the testing and the beginning of lockdown measures or COVID-19 infection: short-term period (1–4 months), medium-term period (5–8 months), and long-term period (9–12 months; see Table 1 and Table 2).

## 3. Results

The results obtained in this review are shown in Table 1 for cognitive studies related to COVID-19 lockdown measures on people with neurological conditions and healthy people, and in Table 2 for cognitive studies related to COVID-19 cognitive sequelae due to COVID-19 infection in healthy people.

As reported in Table 1, we identified the negative effects of COVID-19 lockdown measures on cognitive functions in 12 out of 16 studies. In particular, during the first four months of COVID-19 lockdown measures (short period), a worsening in cognitive performance was reported in four out of seven studies in different neurological populations [25,42,43,44]. In particular, in most of the patients, a decline in cognitive functions resulted from the MMSE, while in Tsatali et al. [44], a worsening in learning and phonemic fluency in people with MCI and AD was reported. Conversely, Dura-Perez et al. [40], Gareri et al. [41] and Vislapuu et al. [45] did not find significant cognitive differences in people with neurological conditions due to COVID-19 lockdown measures. During the medium and long period of COVID-19 lockdown measures, all groups of neurological patients exhibited a significant decline in functional and cognitive status compared to the pre-COVID period. During the medium period (5 to 8 months), three out of four studies showed adverse effects of COVID-19 lockdown measures on attention [46], and on the overall patients’ cognitive status [48,49], except for Ref. [47]. During the long period (9 to 12 months), three studies reported a decrease in the patients’ overall cognitive status [27,50,51].

Only two studies were performed on healthy people by using standardized tests. The study by Pisano et al. [20], performed in the first four months of the lockdown measures (short period), reported a worsening in working and prospective memory performance in a group of 150 college students; while in the medium period, the only study by Favieri et al. [52] showed impaired executive functioning and motor inhibition in a sample of 90 college students.

As reported in Table 2, the negative effects of COVID-19 infection on cognitive performance in healthy people were identified in 39 out of 46 studies (85%). Five out of seven studies performed in the short period (1–4 months) found a general worsening in cognitive performance [53,54,57,58], specifically, in verbal memory [54,57] and attention tasks [55]. On the contrary, Johnsen et al. [56] and Priftis at al. [59] did not find significative differences in any cognitive domains.

A total of 27 out of 32 articles reported negative effects of COVID-19 infection during the medium period (5–8 months). As in the short period, most of the authors found a significant general cognitive decline [60,61,62,63,64,68,69,70,73,77,78,82,83,84,85], in particular, in memory [62,65,66,74,77,81,85,86,87,88], verbal fluency [62,65,66,71,72,88], executive functions [65,69,72,74,75,81,87,88] and attention tasks [65,72,76,89]. Three studies did not report significant effects on cognitive performance in hospitalized people that resulted positive in the SARS CoV-2 nasopharyngeal test compared to those with no history of the virus [37,38,79]; while, in the Pilotto et al. [83] and Stallmach et al. [84] study, a very low percentage of people with COVID-19 infection showed the presence of cognitive decline.

The seven studies which have investigated the long-term effects of COVID infection (9 to 12 months) found a deterioration of cognitive performance in different cognitive domains, such as in overall cognition [91], memory [90,92,93,94], attention [95,96], executive functions [90,92] and visuospatial abilities [90].

## 4. Discussion

This review aims to present a comprehensive overview of the literature related to the effects of lockdown measures and COVID-19 infection on cognitive functioning in healthy and neurological populations. Considering the large number of papers published to date on these topics, as far as we know, this is the first review which investigates the effects of the pandemic on cognitive functioning by using standardized cognitive tests. Indeed, most of the studies have included self-reported measures, such as questionnaires. In clinical practice and/or research investigation, choosing an appropriate cognitive functional measure is first of all a critical decision for the necessity to refer to measures with robust reliability [97]. In general, two main measures, self-reported questionnaires and standardized tests, are used to assess cognitive functioning. Self-reported measures are favored among clinicians and researchers because they are relatively easy to administer and they are time and cost-effective [98,99]. However, it is well known that they are more susceptible to social desirability and self-reported bias [100]. The main disadvantage of self-reported questionnaires might also be the possibility of providing invalid answers. While responding to the items, respondents may not answer truthfully, especially on sensitive questions [101]. Conversely, standardized tests overcome some of these limitations. The main benefit of standardized tests is that they are objective measures, more reliable and valid than non-standardized measures [102]. They often provide some type of “standard score” which can help interpret how far participant’s results range from the average [102]. A recent multilevel random-effects meta-analysis revealed no relationship between self-reported and neuropsychological tests of cognitive flexibility, suggesting that self-reported questionnaires should no longer be considered valid proxies for measuring cognitive flexibility [102]. For these reasons, in the present review, we have decided to include only studies on the impact of COVID-19 lockdown measures or COVID-19 infection on cognitive standardized tests.

Surprisingly, our research revealed that only two works have used standardized tests during COVID-19 lockdown measures in healthy subjects compared to neurological populations. Indeed, during the lockdown, most studies have applied standardized tests in people with neurodegenerative diseases (i.e., MCI, PD, AD). Probably because healthy subjects are considered capable of responding autonomously, researchers have preferred to test them by using self-reported questionnaires that are easily administered online. In contrast, researchers were very much concerned with investigating whether or not, due to the adoption of lockdown measures, neurodegenerative populations presented a worsening in their cognitive status; thus, they chose standardized tests as more reliable measures. In general, almost all studies indicated a decrease in the MMSE and MoCA’s score, two measurements widely adopted in clinical practice to detect the presence of cognitive decline in neurodegenerative diseases as an index of disease progression [39,103,104]. We cannot state unequivocally whether or not this worsening was due to the adoption of confinement measures, or to the characteristics of the disease whose symptoms tend to worsen over time in neurodegenerative populations. It could also be argued that, since several studies have reported higher levels of anxiety and depression in these people [22,105,106], their psychological status has, in turn, contributed to an increase in cognitive decline. Indeed, changes in everyday life routines were applied during the pandemic leading to a worsening in the psychological status of different populations [107]. For instance, since people with dementia usually require daily assistance, they could not have rapidly adapted themselves to changing situations as was required by the pandemic [108]. Thus, the lack of social stimulation and pleasurable activities favored the onset of anxiety and depression, which, in turn, cognitively affected the progression of the disease [106]. During the first wave of COVID-19, together with a general cognitive decline, Aragón et al., 2022 [46] reported a worsening in selective attention tasks in four patients with subjective cognitive decline and forty-seven MCI participants. These tasks were appropriately designed by the authors for testing executive attention. The first task was an audio dictation of reverse digits backwards. The second task included another audio with a song fragment in which patients had to count the number of times they heard a designated word and write the answer with a maximum score of 19 [46].

In terms of the two studies on healthy subjects, Pisano et al. [20] showed a decline in working and prospective memory, measured with the PASAT [109] and the MIST [110] test, in a sample of young university students, while Favieri et al., 2022 [52] reported a decline in executive functions, measured with the STROOP test [111], and in motor inhibition in a Go/No-Go task, in ninety college students.

In contrast, all studies on the effects of COVID-19 infection on cognition, measured through standardized tests, have been conducted on healthy individuals. Indeed, the vast majority of research has intentionally excluded individuals with previous neurological and psychiatric disorders, who would have confounded the interpretation of the results [93]. Almost all studies reported the presence of a general cognitive decline [60,61,62,63,64,68,69,70,73,77,78,82,83,84,85] (see Table 2), which is a common sequela of other viral diseases, such as AIDS [112,113] and sepsis ([114,115]. In the literature, this status is often referred to as ‘Long COVID’ [116,117], or ‘brain fog’ with accompanied clinical symptoms, such as low energy, insomnia, problems in concentration and spatial orientation and difficulty in finding the right words [118]. In particular, some studies reported a decrease in short-and-long term memory performance [62,65,66,74,77,81,85,86,87,88], in verbal fluency [62,65,66,71,72,88], in executive functions [65,69,72,74,75,81,87,88] and in selective attention tasks [65,72,76,89]. It is likely the case, as suggested by previous studies, that these cognitive deficits occurred as a consequence of respiratory symptoms severity due to the pandemic [119,120]. Indeed, cognitive deficits in people who were intubated and/or required a lengthy hospital stay are expected due to the lack of oxygen to the brain [118]. Respiratory viruses manage to bypass the blood–brain barrier using either infected blood cells, such as “Trojan Horses”, or by exploiting the axonal route, crossing neurons one by one [121]. Similarly, in milder cases who have not been hospitalized, it is possible that the lowest cognitive implications were due to less severe hypoxia [118]. Indeed, several studies have suggested that COVID-19 infection may cause alterations in white and grey matter volume of the hippocampus, which plays a central role in learning and memory [122,123,124]. Accordingly, the effects on the hippocampus are due to the hypoxic and hypoxemic conditions of COVID-19 patients, which exert a negative effect on hippocampal neurogenesis [125]. As previously reported, other impaired cognitive domains, reported in healthy people due to COVID-19 infection, were present in selective attention and executive functions tasks [55,57,62,65,71,81,88]. Interestingly, a recent report on a single case neuroimaging study with anosmia, due to COVID-19, revealed reduced metabolic activity in the orbitofrontal cortex, suggesting impaired neural function in this region [126]. It is well-known that the orbitofrontal cortex is responsible not only for the detection of common odors [127], but also for executive functions and attentional processing [128,129,130]. Thus, although future studies should elucidate this issue, the hypothesis might be advanced that, together with the lack of oxygen to the brain due to respiratory symptoms, executive functions and attentional deficits also arise as a consequence of abnormal activity in the orbitofrontal cortex.

It is worth considering that the studies reported in our review on neurological populations revealed the presence of cognitive decline regardless of the time elapsed between the beginning of the confinement measures and the administration of standardized tests. Indeed, the presence of a worsening in cognitive performance in these populations was present independently of the time period in which the tests were performed (short 1–4 months, medium 5–8 months, long 9–12 months; see Table 1). Similarly, studies in healthy subjects revealed the presence of cognitive deficits in the three time periods following COVID-19 infection, albeit most studies tested participants between five to eight months after the infection. As far as we know, this is the first review which investigates the impact of confinement measures and COVID-19 infection in neurological and healthy populations by including only standardized cognitive tests. The pandemic has been an unexpected, dramatic event that spread panic among civilians and insecurity at all socio-political and economic levels, suddenly disrupting everyday life. Thus, it was expected that it would immediately impact the population as a whole with severe psychological and cognitive implications. Indeed, our findings are in line with previous literature on COVID-19 which report the presence of cognitive decline in the short [20,42,53], medium [49,52,61], and long-term periods [50,92].

## 5. Conclusions

In conclusion, our review is the first to highlight the importance of considering standardized tests as reliable measures to quantify the presence of cognitive deficits due to COVID-19. Indeed, we strongly believe that these tests guarantee a valid, objective measure of the cognitive status tested in various populations. By administrating the same test over time, clinicians and researchers have the main advantage to show significant changes referring to the same normative data. In addition, patients’ test scores can also be easily compared to each other to identify the presence of cognitive difficulties in a particular area, thus, allowing clinicians for the planning of rehabilitation treatments focused on the impaired cognitive domain. This choice could be of great help to many patients who still, nowadays, experience post-COVID-19 symptoms.

## Figures and Tables

**Table 1:** Summary of studies reporting the negative effects of COVID-19 lockdown measures on cognitive performance, respectively, in people with neurological conditions and healthy people for the three-time testing periods (short 1–4 months, medium 5–8 months, long 9–12 months).

Author/s	Location	Participants	Time Elapsed between the Testing and the Beginning of Confinement Measures	Standardized Tests	Cognitive Outcomes
Baschi et al., 2020 [25]	Italy	PD N = 96 N = 96 carers	Short	Itel-MMSE;	Both groups showed a worsening of pre-existing cognitive symptoms (37.5%), and new behavioural (26%), and motor symptoms (35.4%) during the COVID-19 lockdown, resulting in an increased caregiver burden in 26% of cases.
Dura-Perez et al., 2022 [40]	Spain	MCI N = 151	Short	MMSE	The outbreak did not significantly impact cognition in comparison with baseline assessments prior to the outbreak.
Gareri et al., 2022 [41]	Italy	MCI N = 4 Vascular Dementia N = 30 AD = N = 28 Mixed Dementia N = 19 Frontotemporal Dementia N = 6 PD N = 2 Dementia with Lewy Bodies N = 1	Short	MMSE	Most of the patients were clinically stable over time.
Paolini et al., 2021 [42]	Italy	MCI N = 38	Short	MMSE;ENB-2	Cognitive functioning worsened during the lockdown.
Tondo, Sarasso, Serra, Tesser and Comi, 2021 [43]	Italy	AD N = 68 Vascular Dementia N = 28 MCI N = 23 Frontotemporal Dementia N = 9 Lewy Bodies Dementia N =4	Short	MMSE	The 2020-GROUP showed a significant loss of MMSE points per year compared to the 2019-GROUP and the 2018-GROUP (*p* = 0.021).
Tsatali et al., 2021 [44]	Greece	MCI N = 296 AD N = 111	Short	MMSE; MoCA; RAVLT; Phonemic Fluency; ROCF; WAIS	During the lockdown period, MCI and AD patients’ neuropsychological performance did not change (MMSE and MoCA), except for verbal memory (RAVLT), learning (WAIS), and phonemic fluency.
Vislapuu et al., 2021 [45]	Norway	Dementia N = 105 N = 105 carers	Short	MMSE	Higher cognitive function (*p* = 0.044) was associated with a reduction in home nursing service during the lockdown.
Aragón et al., 2022 [46]	Argentina	MCI N = 47	Medium	Verbal fluency task; Memory task; Attention task; Reverse Digits	Performance worsened only in the Selective Attention Task.
Custodio et al., 2021 [47]	Peru	AD N = 91 N = 91 carers	Medium	RUDAS; M@T; CDR	No significant differences were found in overall cognition (RUDAS), memory (M@T) and dementia severity (CDR) scores.
Pereiro et al., 2021 [48]	Spain	N = 98 Unspecified Neurological	Medium	MMSE; CDR	Lower cognitive (MMSE) and functional scores (CDR) resulted during the lockdown compared to pre-COVIDE-19 time.
Tsiakiri, Vlotinou, Terzoudi, Heliopoulos and Vadikolias, 2022 [49]	Greece	MCI N = 34 Dementia N = 21 N = 70 controls	Medium	MMSE; MoCA	In the patients‘ group, cognitive performance worsened with respect to the pre-COVIDE-19 time (MMSE and MoCA).
Chen et al., 2021 [27]	China	MCI N = 50 AD N = 105 Lewy Bodies Dementia N = 22	Long	MoCA; MMSE; NPI	42% of MCI, 54.3% of AD and 72.7% of DLB showed a decline in MMSE scores and 54.4% of DLB reported a worsening in the neuropsychiatric inventory (NPI) scores. DLB showed a more rapid decline in the MMSE than AD.
Gan et al., 2021 [50]	China	AD N = 131 Unspecified Dementia N = 60 MCI N = 14	Long	C-MMSE; MoCA; CDR	A worsening in cognitive performance was reported in the MMSE and MoCA and in the NPI with respect to the pre-COVID-19 time.
Vernuccio et al., 2022 [51]	Italy	AD N = 34 MCI N = 28 Mixed Dementia N = 20 Vascular Dementia N = 13 PD N = 2 Frontotemporal Dementia N = 2 Lewy Bodies Dementia N = 1	Long	MMSE	A significant functional and cognitive decline was observed during the lockdown compared to the pre-COVID-19 time.
Pisano et al., 2021 [20]	Italy	N = 150 Healthy People	Short	PASAT; MIST	A significant decrease in the participants’ working memory (PASAT) and in prospective memory (MIST) was present during the lockdown period compared to normative data.
Favieri et al., 2022 [52]	Italy	N = 90 Healthy People	Medium	Stroop Test; Go/No-Go Task	Impaired Executive Functioning (Stroop Test) and in Motor Inhibition (Go/No-Go Task) was found in people with higher post-traumatic stress symptoms.

Legend. AD: Alzheimer’s Disease; PD: Parkinson’s Disease; MCI: Mild Cognitive Impairment; MMSE: Mini Mental State Examination; ENB: Esame Neuropsicologico Breve; MoCA: Montreal Cognitive Assessment; RAVLT: Rey Auditory Verbal Learning Test; ROCF: Rey–Osterrieth Complex Figure; WAIS: Wechsler Adult Intelligence Scale; RUDAS: Rowland Universal Dementia Assessment Scale; M@T: Memory Alteration Test; CDR: Clinical Dementia Rating; PASAT: Paced Auditory Serial Addition Task; MIST: Memory for Intentions Test.

**Table 2:** Summary of studies reporting the negative effects of COVID-19 infection on cognitive performance in healthy people for the three time periods (short 1–4 months, medium 5–8 months, long 9–12 months).

Author/s	Location	Participants	Time Elapsed between the Testing and the Beginning of COVID-19 Infection	Standardized Tests	Cognitive Outcomes
Cacciatore et al., 2022 [53]	Italy	N = 83 Healthy People	Short	MoCA;	The average MoCA score revealed a worsening in cognitive performance.
Cian, De Laurenzis, Siri, Gusmeroli & Canesi, 2022 [54]	Italy	N = 29 Healthy People N = 29 matched controls	Short	MMSE; RAVLT; CPM47; CDT; Phonemic/semantic and alternate fluency; Digit Span Forward and Backward	Significant differences between groups with and without COVID-19 (control) were found in the memory subtests (immediate, recall and recognition, RALVT). The MMSE, logical reasoning (CPM,) digit forward and backward, phonemic, semantic, and alternate fluency and executive functioning (CDT) did not show the presence of cognitive decline.
do Carmo Filho, van Duinkerken, Tolentino and Schmidt, 2022 [55]	Brazil	N = 30 Healthy People N = 30 matched controls	Short	CVAT	Attentional performance (CVAT) was significantly worse in COVID-19 survivors when compared with controls and test norms.
Johnsen et al., 2021 [56]	Denmark	N = 57 Healthy People	Short	SCIP-D; TMT	The SCIP-D did not reveal the presence of cognitive decline and or attention deficits (TMT).
Méndez et al., 2021 [57]	Spain	N = 179 Healthy People	Short	SCIP; FAS; WAIS-III	38% of participants presented moderate impairment and 11.2% severe impairment in immediate verbal memory task (SCIP). In relation to delayed memory, 11.8% reported moderate and 2.8% severe impairment (SCIP). In semantic verbal fluency, 34.6% showed moderate and 8.4% severe deficits (FAS). Working memory was moderately impaired in 6.1% and severely impaired in 1.1% participants (WAIS-III). Finally, 105 (58.7%) participants met criteria for moderate and 33 (18.4%) for severe cognitive impairment.
Pistarini et al., 2021 [58]	Italy	N = 20 Healthy People	Short	MMSE; MoCA	Results showed that 35% of the participants manifested cognitive decline in the MMSE and in the MoCA.
Priftis et al., 2022 [59]	Italy	N = 22 Healthy People	Short	MMSE; Corsi Backward and Forward; Digit Span Forward and Backward; RAVL; Semantic and phonemic fluency; TMT; Stroop Test, WCST	In total, 93.2% of the participants performed normally in phonological working memory task (digit span); 90.9% in long-term verbal learning (RAVL); 95.5% in visuospatial perception and praxis; and 82% in visuospatial long-term learning. On average, 96% performed normally also in attention and executive functions tasks (TMT, WCST, Stroop test).
Birberg Thornberg et al., 2022 [60]	Sweden	N = 133 Healthy People	Medium	RBANS	In the RBANS global cognition index (attention, language, short-term memory, visuospatial abilities), 60% performed under the cut-off scores.
Braga et al., 2022 [61]	Brazil	N = 614 Healthy People	Medium	BNIS	The BNIS revealed the presence of cognitive decline in memory tasks.
Calabria et al., 2022 [62]	Spain	N = 136 Healthy People	Medium	T-MoCA; CPT-II; RAVLT; ROCF; BNT; Digit Span Forward and Backward; Block Design; Symbol Search; TMT; Stroop Test	95 participants (69.8%) showed the presence of cognitive decline (T-MoCA), 6 (4.4%) were impaired in naming (BNT), 25 (18.3%) in semantic fluency, 23 (16.9%) in phonological fluency, and 44 (32.3%) in memory tasks (RAVLT, ROCFT). All patients showed difficulties in the attention task (CPT-II) and approximately 25% in executive functioning (Stroop test, TMT).
Costas-Carrera et al., 2022 [63]	Spain	N = 58 Healthy People	Medium	MoCA; Digit Span Forward and Backward; WAIS-III; Stroop Test; FCSRT; JLO; TMT; COWAT; ANF; BNT	53.4% of participants revealed the presence of mild cognitive impairment (MOCA). Compared to clinical data, on average all participants performed above cut-off scores in all other tests.
Cristillo et al., 2022 [64]	Italy	N = 106 Healthy People	Medium	MoCA	18 participants (17.82%) reported MoCA scores below the cut-off.
Crivelli et al., 2022 [65]	Argentina	N = 45 Healthy People N = 45 matched controls	Medium	MoCA; TMT; Digit Span Forward; DSC; Craft Story;RAVL; BFT; WCST; Stroop Test; Phonological fluency; Semantic fluency;CDT; MNT	Compared to healthy controls, COVID-19 subjects reported a worse performance in memory tests (RAVLT, Digit span), naming (BNT), semantic and phonemic fluency, attention, and executive functions (TMT-A, TMT-B, WCST, CDT).
Dondaine et al., 2022 [66]	France	N = 62 Healthy People	Medium	FCSRT; WAIS-IV;CPT3;Categorical and fluency test; TMT	Approximately 25% of participants reported pathological scores in memory tests (FCSRT), 11% in digit span, 6% in phonemic and semantic fluency and 17% in sustained attention (CPT3).
Dressing et al., 2022 [67]	Germany	N = 31 Healthy People	Medium	HVLT; BVMT-R; Digit Span Forward and Backward; TMT; FWIT; SMDT; Semantic and phonemic fluency; MoCA	The MoCA did not reveal the presence of cognitive decline and, in general, half of the participants (N = 16) performed above the cut-off scores in all tests.
Duindam, Kessels, van den Borst, Pickkers and Abdo, 2022 [68]	Netherlands	N = 96 Healthy People	Medium	MoCA; TMT; LDST; Digit Span	26 participants (27%) were classified as cognitively impaired based on their test results. More specifically, 5% showed cognitive decline in the MoCA. On executive functioning tests, 21% were impaired in the TMT-A/B, and 18% in Digit Span test. Information-processing performances (LDST and TMT-A) were impaired in 23% and 15% of participants, respectively.
Ferrucci et al., 2021 [69]	Italy	N = 38 Healthy People	Medium	BRB-NT	42% showed processing speed deficits, 26% delayed verbal recall deficits and 10% immediate verbal recall deficits. Visual long-term and short-term memory were impaired in 18% and 16%, respectively. Working memory and semantic verbal fluency were impaired in 10% and 8% of participants, respectively.
Frontera et al., 2021 [37]	USA	N = 196 Healthy People N = 186 controls	Medium	t-MoCA	Cognitive metrics were similar between the COVID-19 and control groups.
García-Grimshaw et al., 2022 [70]	Mexico	N = 92 Healthy People	Medium	MoCA	The overall mean MoCA total scores were below the cut-off.
García-Molina et al., 2022 [71]	Spain	N = 91 Healthy People N = 32 controls	Medium	BT; WAIS-III; RAVLT; Spanish-language neuropsychological battery	Significant differences were present between groups in learning, recall and recognition of the memory subtests (RAVLT), and in verbal fluency.
García-Sánchez et al., 2022 [72]	Spain	N = 63 Healthy People	Medium	MoCA; CPT-II; RAVLT; ROCF; Digit Span Forward and Backward; BNT; Block Design; Coding; Symbol Search; TMT; Stroop Test; Verbal fluency tasks; 15-Objects Test	19% of participants were impaired in Attention (TMT), 5% in executive functioning (TMT, Stroop test), 9.5% in long-term memory (RAVLT), 5 % in short-term memory (digit span) and 1.6% in naming (BNT).
Hadad et al., 2022 [73]	Israel	N = 46 Healthy People	Medium	MoCA	Compared to normative data, all participants were below the cut-off score in the MoCA showing the presence of cognitive decline.
Hampshire et al., 2022 [74]	UK	N = 46 Healthy People N = 460 matched controls	Medium	Cognitron	Compared to matched controls, participants were significantly less accurate in verbal analogies, 2D manipulation, verbal, and spatial short-term memory tests.
Holdsworth et al., 2022 [38]	UK	N = 205 Healthy People	Medium	NIH Toolbox	The assessment of different cognitive functions (language, executive functioning, episodic and working memory) revealed normal performance.
Krishnan, Miller, Reiter and Bonner Jackson, 2022 [75]	USA	N = 20 Healthy People	Medium	WMS-IV; RAVLT; BMVT-R; WRAT-IV; BNT; Semantic and phonemic fluency; JLO; WAIS-IV; DKEFS; TMT; WCST; CPT-3; SDMT	20% of participants showed impairment in executive functions (TMT, WCST) and in the visuospatial Memory Test.
Lamontagne, Winters, Pizzagalli and Olmstead, 2021 [76]	USA	N = 50 Healthy People N = 50 controls	Medium	ANT	COVID-19 participants reported a worsening in attention performance (ANT) compared to the control group.
Lier et al., 2022 [77]	Germany	N = 105 Healthy People N = 55 controls	Medium	MoCA; TMT; Semantic fluency	35 % of the participants showed slight cognitive impairments in the MoCA; deficits were also detected in memory, letter fluency and visuospatial functions (TMT); semantic verbal fluency was impaired in 14%.
Lynch et al., 2022 [78]	USA	N = 60 Healthy People	Medium	MoCA; RBANS; TMT; Verbal fluency; Stroop test; TOPF	36.7% showed the presence of cognitive decline in the MoCA.
Mattioli et al., 2021 [79]	Italy	N = 120 Healthy People N = 30 controls	Medium	MMSE; COWA; ROCF; CVLT; TEA; TOL	No significant differences between the group with COVID-19 and the group without COVID-19 were found in any of the tests used.
Mattioli et al., 2022 [80]	Italy	N = 215 Healthy People	Medium	MMSE; COWA-S; COWA-Ph; ROCF; CVLT; RAVLT; TOL	No significant differences between the group with COVID-19 and the group without COVID-19 were found in any of the tests used.
Miskowiak et al., 2021 [81]	Denmark	N = 29 Healthy People N = 100 matched controls	Medium	SCIP-D; TMT-B	When compared to controls, participants had a significantly worse performance in Verbal Learning and Working Memory subtests of SCIP-D. Compared to norms, executive functioning (TMT-B) was also impaired.
Ortelli et al., 2022 [82]	Italy	N = 67 Healthy People N = 22 matched controls	Medium	MoCA; FAB; Sustained Attention Task; Stroop Test; Navon Task	Compared to controls, significant differences were present in all tests in the COVID-19 group indicating the presence of cognitive decline (MoCA), in executive functions and sustained attention.
Pilotto et al., 2021 [83]	Italy	N = 165 Healthy People	Medium	MoCA	Only 10% of participants showed the presence of cognitive decline in the MoCA test.
Stallmach et al., 2022 [84]	Germany	N = 355 Healthy People	Medium	MoCA	Only 21% of participants showed the presence of cognitive decline in the MoCA test.
Vannorsdall et al., 2021 [85]	USA	N = 82 Healthy People	Medium	RAVLT; TMT; Digit span forward and backward; Phonemic and semantic fluency; verbal fluency	Post-COVID-19 clinic patients produced lower cognitive scores than non-COVID-19 patients.
Voruz et al., 2022 [86]	Switzerland	N = 102 Healthy People	Medium	Stroop test; TMT;GREFEX; Grober and Buschke free/cued recall paradigm; Digit Span Backward; Corsi backward; TAP; Digit Span Forward; ROCF; BECLA; MEM-III; VOSP; WAIS-IV; GERT	Analyses revealed that anosognosic participants (N = 26 who were not conscious about their memory deficits) performed more poorly than nosognosic participants (N = 76 who were conscious about their memory deficits) in verbal episodic memory (Grober and Buschke free/cued recall paradigm), visuospatial episodic memory (Rey figure), verbal short-term memory (MEM-III) and in Mental flexibility (GREFEX).
Voruz et al., 2023 [87]	Switzerland	N = 121 Healthy People	Medium	VOSP; Moroni Praxis Battery; BECLA; GREFEX; WMS-III; WAIS-IV; TAP; ROCF; SAD	Significantly different performances in executive functioning (GREFEX) and in memory tests (WMS-III, ROCF).
Whiteside et al., 2022 [88]	USA	N = 49 Healthy People	Medium	WAIS-IV; COWAT; Animal Fluency; Grooved Pegboard Test HVLT-R; WCST; Stroop Test; TMT	Impaired performances in Working Memory (WAIS-IV), Memory (HVLT-R; ROCF) and Executive Functioning (WCST; Stroop).
Zhao et al., 2022 [89]	UK	N = 53 Healthy People N = 83 matched controls	Medium	Sustained Visual Attention Task	In the COVID-19 group, accuracy resulted more impaired than in the control group, but no differences were present in reaction times.
Andriuta et al., 2022 [90]	France	N = 46 Healthy People N = 1003 matched controls	Long	MMSE; BNT; ROCF; FCSRT; DPT; GREFEX Verbal fluency test; TMT; Stroop Test	The COVID-19 group showed a deterioration in language (GREFEX, BNT), executive functioning (TMT; Stroop Test) and memory (MMSE; ROCF).
Cristillo et al., 2022 [91]	Italy	N = 132 Healthy People	Long	MoCA	Logistic regression showed a significant correlation between brain fog and the self-rating depression scale values (*p* = 0.020).
Delgado-Alonso et al., 2022 [92]	Spain	N = 50 Healthy People N = 50 matched controls	Long	Digit Span Forward and Backward; Corsi test; SDMT; BNT; JLO; ROCF; FCSRT; Verbal Fluency; Stroop Test; VOSP; TMT; FGT; TOL-F; INHIB; N-Back Verbal Test; Cognitrone; WAF	Participants reported significantly worse performance compared to matched controls in Memory (FGT), Executive Functioning (TMT-A; TMT-B), and Visuospatial abilities (WAF).
Díez-Cirarda et al., 2022 [93]	Spain	N = 86 Healthy People N = 36 controls	Long	Digit Span Forward and Backward; SDMT; FCSRT; ROCF; Verbal Fluency; Stroop Test; BNT; JLO; VOSP	Most cognitive alterations were detected in attention (SDMT) and working memory (digit span) (up to 44.2%), but deficits were also found in memory (FCSRT) (up to 40.7%) and executive functions (Stroop test) (up to 39.5%), followed by visuospatial ability (JLO) (up to 36%), and naming (BNT, verbal fluency) (up to 18.6%).
Fiorentino et al., 2022 [94]	France	N = 84 Healthy People	Long	PPTT; Grémots battery;	Semantic memory was impaired in 17 participants (20%).
Jennings, Monaghan, Xue, Duggan and Romero-Ortuño, 2022 [95]	Ireland	N = 108 Healthy People	Long	Simple Response Time; Choice Reaction Time	Participants with self-reported brain fog had higher mean reaction time in simple response time (*p* = 0.028) and in choice reaction time (*p* = 0.035).
Santoyo-Mora et al., 2022 [96]	Mexico	N = 106 Healthy People N = 38 matched controls	Long	2AFC Test; Simple Reaction Test	Compared to controls, participants recovered from a severe–critical COVID-19 infection showed a poor performance in different cognitive tests: decision-making tasks (2AFC) and information processing speed.

Legend. MoCA: Montreal Cognitive Assessment; MMSE: Mini Mental State Examination; RAVLT: Rey Auditory Verbal Learning Test; CPM47: Coloured Progressive Matrices 47; CDT: Clock Drawing Test; CVAT: Continuous Visual Attention Test; SCIP: Screen for Cognitive Impairment in Psychiatry; TMT: Trail Making Test; FAS: F-A-S Test; WAIS: Wechsler Adult Intelligence Scale; WCST: Wisconsin Card Sortin Test; RBANS: Repeatable Battery for the Assessment of Neuropsychological Status; BNIS: Barrow Neurological Institute Screen for Higher Cerebral Functions; CPT: Continuous Performance Test; ROCF: Rey–Osterrieth Complex Figure; BNT: Boston Naming Test; FCSRT: Free and Cued Selective Reminding Test; JLO: Judgment of Line Orientation; COWAT: Controlled Oral Word Association Test; ANF: Animal Fluency Test; DSC: Digit-Symbol Coding; BFT: Benson Figure Test; MNT: Multilingual Naming Test; HVLT: Hopkins Verbal Learning Test; BVMT-R: Brief Visuospatial Memory Test-Revised; FWIT: Colour-Word Interference Test; SMDT: Symbol-Digit Modalities Test; LDST: Letter Digit Substitution Test; BRB-NT: Brief Repeatable Battery of Neuropsychological Tests; BT: Barcelona Test; DKEFS: Delis–Kaplan Executive Function System; ANT: Attention Network Test; TOPF: Test of Premorbid Functioning; CVLT: California Verbal Learning Test; TEA: Test of Everyday Attention; TOL: Tower of London; FAB: Frontal Assessment Battery; BECLA: Batterie d’Évaluation Cognitive du Langage; MEM: Échelle clinique de mémoire de Wechsler; GERT: Geneva Emotion Recognition Test; VOSP: Visual Object and Space Perception; TAP: Test for Attentional Performance; SAD: Self-Appraisal Discrepancy; DPT: Doors and People Test; FGT: Figural Memory Test; WAF: Perception and Attention Functions; PPTT: Pyramids and Palm Trees Test; 2AFC: Two-Forced Alternative Choice.

## Data Availability

Data sharing not applicable.
